# The relationship between ciliary neurotrophic factor (CNTF) genotype and motor unit physiology: preliminary studies

**DOI:** 10.1186/1472-6793-5-15

**Published:** 2005-09-23

**Authors:** Robin A Conwit, Shari Ling, Stephen Roth, Daniel Stashuk, Ben Hurley, Robert Ferrell, E Jeffrey Metter

**Affiliations:** 1 National Institute on Neurological Disorders and Stroke, Rockville, MD, USA 20892; 2 Clinical Research Branch, National Institute on Aging, National Institute on Aging Intramural Research Program, Harbor Hospital, 5^th ^Floor, Baltimore, MD, USA 21225; 3 Department of Kinesiology, University of Maryland, College Park, MD, USA; 4 University of Waterloo, Department of Systems Design Engineering, Waterloo, Ontario, Canada; 5 Department of Human Genetics, University of Pittsburgh, Pittsburgh, PA, USA

## Abstract

**Background:**

Ciliary neurotrophic factor (CNTF) is important for neuronal and muscle development, and genetic variation in the CNTF gene has been associated with muscle strength. The effect of CNTF on nerve development suggests that CNTF genotype may be associated with force production via its influence on motor unit size and firing patterns. The purpose of this study is to examine whether CNTF genotype differentially affects motor unit activation in the vastus medialis with increasing isometric force during knee extension.

**Results:**

Sixty-nine healthy subjects were genotyped for the presence of the G and A (null) alleles in the CNTF gene (n = 57 G/G, 12 G/A). They were tested using a dynamometer during submaximal isometric knee extension contractions that were from 10–50% of their maximal strength. During the contractions, the vastus medialis was studied using surface and intramuscular electromyography with spiked triggered averaging to assess surface-detected motor unit potential (SMUP) area and mean firing rates (mFR) from identified motor units. CNTF genotyping was performed using standard PCR techniques from DNA obtained from leucocytes of whole blood samples. The CNTF G/A genotype was associated with smaller SMUP area motor units and lower mFR at higher force levels, and fewer but larger units at lower force levels than G/G homozygotes. The two groups used motor units with different size and activation characteristics with increasing force generation. While G/G subjects tended to utilize larger motor units with increasing force, G/A subjects showed relatively less increase in size by using relatively larger units at lower force levels. At higher force levels, G/A subjects were able to generate more force per motor unit size suggesting more efficient motor unit function with increasing muscle force.

**Conclusion:**

Differential motor unit responses were observed between CNTF genotypes at force levels utilized in daily activities.

## Background

Sarcopenia, the progressive loss of strength and muscle mass with increasing age, is an important contributor to frailty, poor mobility function, and mortality [1,2]. Neuromuscular alterations, and specifically changes in the peripheral nerve, motor unit, and muscle composition, are likely contributors to sarcopenia [3]. Genetic contributions are being explored for both frailty and sarcopenia [4-6].

We recently determined that heterozygotes for the ciliary neurotrophic factor (CNTF) "null" A allele exhibited significantly higher knee extensor concentric (shortening) isokinetic (3.14 rad/s) peak torque, as well as significantly higher knee extensor concentric (shortening) muscle quality (strength per unit of muscle), than G/G homozygotes [4]. The null A allele results in a truncated and presumably inactive mutant CNTF protein [5]. CNTF is a neurotrophic factor important for neuronal and muscle development and growth [7]. The observation of strength differences by CNTF genotype and the known role of CNTF on nerve development suggest that genetic differences are likely to impact on neuromuscular organization by altering muscle, nerve or both and may impact on the development of sarcopenia [8]. These effects may occur by altering the size and firing patterns of motor units, the fundamental organizational unit for muscle activation. We are unaware of any studies that have examined whether CNTF genotype differences impact on motor unit activation in humans.

Muscle force generation is dependent on the number of active motor units and the rate at which they discharge, with recruitment similar to muscle fiber recruitment based on Henneman's size principle [9], i.e. recruited in an orderly sequence according to size, with smaller units activated before larger units. As more motor units are recruited or as motor unit firing rates increase the force of contraction increases. The force produced by a motor unit is dependent on its cross-sectional area, its innervation ratio and the specific tension of its fibers [10]. Different muscles use different strategies to increase force production. Smaller muscles tend to recruit a motor unit, increase its firing rate and subsequently recruit additional units. Larger muscles tend to progressively recruit motor units and only at high force levels increase firing rates.

Clinical techniques have been developed to study motor unit activity during muscle activation [11,12]. The focus of these methods has been on estimating the number of motor units within a muscle. In previous work, we have modified a method developed by Stashuk [13] that decomposed an intramuscular needle EMG signal to identify individual motor units and used spike-triggered averaging of the surface electromyogram (SEMG) to estimate the surface projection of the unit as the surface-detected motor unit potential (SMUP) to examine the distribution of motor unit size and firing rates in the vastus medialis during submaximal isometric contractions of the knee extensors. In previous work examining the vastus medialis [14,15], we found that sampling 15 motor units at force levels corresponding to 10% and 20% of isometric knee extensor maximal voluntary contraction gave a test-retest coefficient of variation of approximately 10%, with test-retest correlations between trials above 0.65 for most comparisons. As muscle force increased during isometric knee extension, the average size of measured motor units increased consistent with an orderly recruitment. Mean firing rate showed only a modest increase up to 30% of maximal voluntary contraction (mean firing rate increasing from 10.1 to 10.8 Hz). A nonlinear relationship was found between muscle force generation and motor unit size with an explained variance of 0.67. We found that the force generated during knee extension was directly related primarily to the size of active motor units and secondarily to firing rate [15].

In the present study, we analyzed data from two studies that included participants in whom CNTF genotype was determined, and motor unit function was assessed. Given the apparent influence of CNTF genotype on muscle strength [4], we hypothesized that subjects with G/A genotype would demonstrate a different motor unit activation pattern with increasing muscle force generation in the vastus medialis than homozygotes with G/G genotype.

The primary focus of this study was to determine the relationship between motor unit size and firing rate in the vastus medialis during knee extensor muscle isometric force generation by sampling the active motor unit pool using the spike triggered averaging and decomposition of needle EMG and SEMG. The primary measures resulting from this approach are an estimate of individual motor unit size from the SMUP and individual motor unit mean firing rates (mFR). Using these direct measures several calculated measures were created to explore the relationship between motor unit size, mean firing rate and muscle isometric force generation (Table 1). In previous work, we have observed that the direct motor unit size measurements, SMUP amplitude and SMUP area can be used to estimate motor unit size, and are highly correlated (r= 0.97) [14], SMUP area provides some advantages when considering the ratios described below. The firing rate is represented by the mFR for the motor unit during the measured part of the contraction. In addition, the product of SMUP area and mFR was calculated and termed the motor unit mean voltage (MUmV). The MUmV represents the average contribution of a motor unit to the acquired surface EMG signal as well as to the resulting generated force, when ignoring contributions by twitch potentiation, nonlinear summation, and other related factors [14]. Jabre and Salzsieder [16,17] defined a similar property that they designated as electrotwitch that was calculated from the instantaneous motor unit firing rate times the macro motor unit potential area and was related directly to the instantaneous force production by a muscle. The MUmV reflects the property that a motor unit increases force production as the firing rate increases to a point where further increases in firing rate have no effect. The average force produced by a muscle is directly related to the product of the mean SMUP area and mean mFR of a representative sample of motor units or in other words by the mean MUmV[15].

To examine the extent of muscle activation, the surface SEMG was rectified, and an average value was obtained over the 30-second contraction. It represents the average electrical activity of the muscle measurable by the electrode during the specific contraction. Using the SEMG, motor unit properties can be expressed relative to the average measured activity of the muscle which allows for the estimation of several properties of the contracting muscle (See Table 1 for a list of terms). During a fatigue study [18], we previously developed and published an index of the number of motor units active during the contraction that can be calculated by dividing the SEMG by the mean MUmV hereafter referred to as the motor unit relative index (MURI). MURI is an estimate of the relative number of motor units being measured by the active recording electrode. SEMG reflects the activity of the whole muscle, while the mean MUmV represents the average motor unit activity during the contraction. MURI is an index and not a direct estimate of the active units, since the ratio does not account for synchronization, nonlinear summation and other factors that could affect the relationship between size and activity. The ratio has some validity, as Suzuki et al [19] found that an increase in mean MUmV was directly related to the relative increase in the SEMG with force generation at increasing percentage of maximal voluntary contraction (MVC).

A second measure is the ratio of motor unit size (mean SMUP area) to force generation, which estimates the average motor unit size per Newton force (N) produced by the knee extensors. The force produced by a single motor unit is dependent on the cross-sectional area of its muscle fibers and the specific tension of its muscle fibers [10]. Presumably, as more motor units are recruited with higher levels of force generation, the type of motor unit will gradually change from the less fatigable type I units to the larger type IIa and IIb units that are capable of generating more force. As this occurs, the force generated per unit of motor unit size should increase to the extent that type II motor units are capable of generating greater force than type I motor units.

A third measure is a ratio of MURI to force which estimates the average number of motor units per N within the field of the surface electrode. This provides an estimate of the force capabilities of the motor units per unit size.

In this preliminary study, we demonstrate that subjects who are heterozygous for the null A allele (i.e. G/A genotype) as compared to subjects homozygous for the common G allele (i.e. G/G genotype) activate motor units with different size and activation characteristics with increasing force generation in the range of 10% to 50% of their maximal knee extensor isometric strength. While G/G subjects tended to utilize larger motor units with increasing force, G/A subjects showed relatively less increase in size by using relatively larger units at lower force levels. At higher force levels, G/A subjects were able to generate more force per motor unit size suggesting more efficient motor unit function with increasing muscle force.

## Results

The subjects included 57 (83%) with the CNTF G/G and 12 (17%) with the G/A genotypes, with no A/A homozygotes represented. These genotype frequencies are similar to those reported previously [4] and the genotype frequencies were in expected Hardy Weinberg equilibrium. Subjects' characteristics are shown in Table 2. No difference or interaction with gender was found between G/A and G/G subjects for MVC, age, height, or weight.

The relationships between motor unit properties and CNTF genotype were examined using mixed effects models adjusted for percent of MVC, force generated, age and gender. The models included an interaction between CNTF genotype and force to test whether the motor unit size utilized by subjects with the G/G and G/A genotypes differed based on force level. The statistical significance levels for direct and calculated motor unit measures are presented in Table 3.

**Figure 1:**
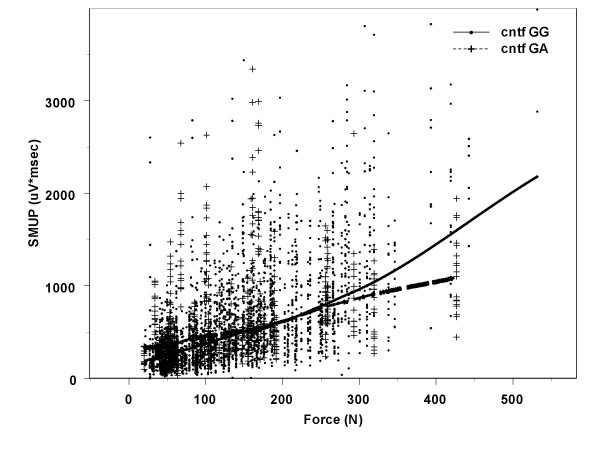
**Relationship between knee extension force and motor unit size (SMUP area) for CNTF GG and GA genotypes. **Data represent individual measurements with a loess regression line for each genotype. Statistical analyses are given in Table 3.

**Figure 2:**
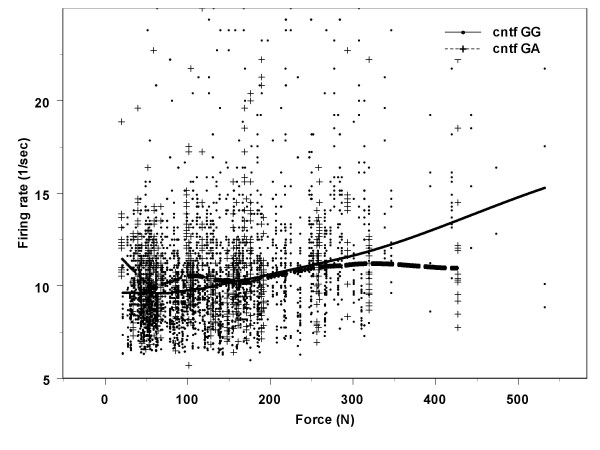
**Relationship between knee extension force and mean motor unit firing rate (mFR) for CNTF GG and GA genotypes. **Data represent individual measurements with a loess regression line for each genotype. Statistical analyses are given in Table 3.

**Figure 3:**
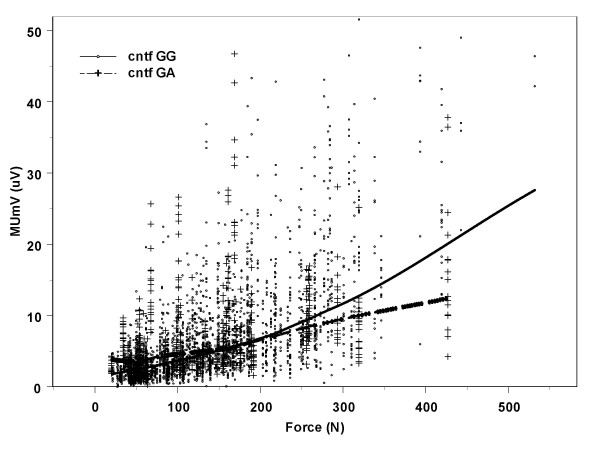
**Relationship between knee extension force and average motor unit contribution considering motor unit size and firing rate (MUmV) for CNTF GG and GA genotypes. **Data represent individual measurements with a loess regression line for each genotype. Statistical analyses are given in Table 3.

**Figure 4:**
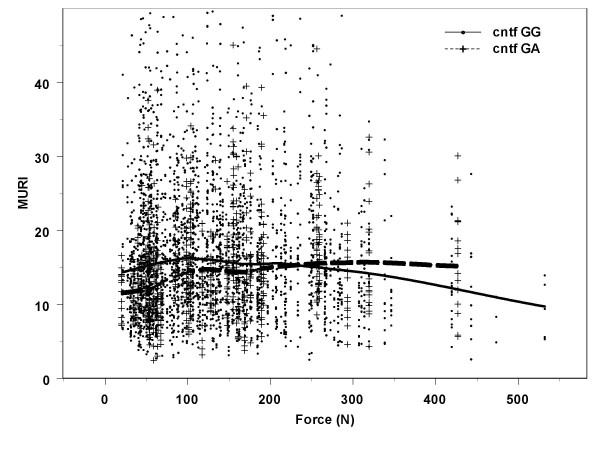
**Relationship between knee extension force and the relative number of motor units active (MURI) for CNTF GG and GA genotypes. **Data represent individual measurements with a loess regression line for each genotype. Statistical analyses are given in Table 3.

**Figure 5:**
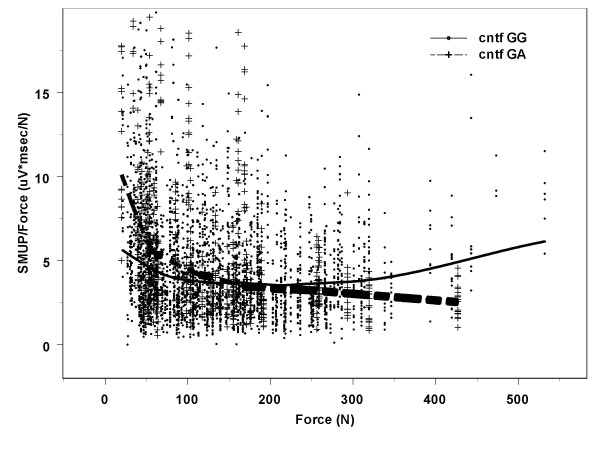
**Relationship between knee extension force and motor unit size per unit force (SMUP area/force) for CNTF GG and GA genotypes. **Data represent individual measurements with a loess regression line for each genotype. Statistical analyses are given in Table 3.

**Figure 6:**
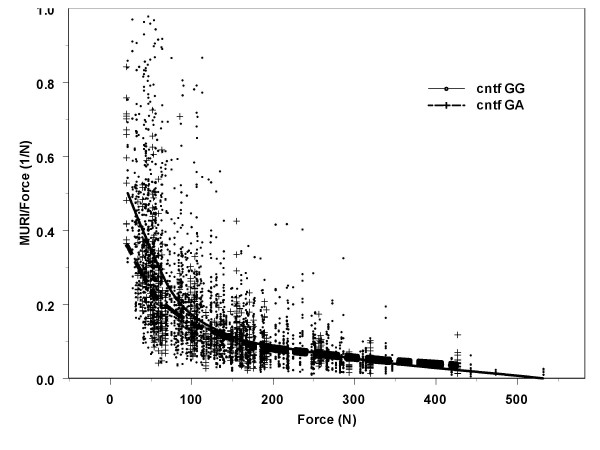
**Relationship between knee extension force and relative number of motor units active per unit force (MURI/force) for CNTF GG and GA genotypes. **Data represent individual measurements with a loess regression line for each genotype. Statistical analyses are given in Table 3.

Significant differences by CNTF genotype and the interaction between CNTF genotype and muscle force generation were present in essentially all models while controlling for age and gender. Only firing rate was not associated with CNTF genotype. The accompanying figures (Figures 1, 2, 3, 4, 5, 6) show the relationships between the motor unit parameter and force generation without adjustments. The figures do not directly reflect the statistical models as the regression lines are based on loess nonparametric smoothing regression which help determine the shape of the regression relationship[20]. The figures show the clear interaction between the EMG measures and muscle force generation between GA and GG CNTF genotypes with an intersection at approximately 200 N.

To address whether the motor units differed in size at lower force levels (< 200 N) and at higher force levels (>= 200 N), separate analyses were examined for both force levels. CNTF genotype and an interaction between CNTF genotype and force were significant when examining only force levels lower than 200 N and greater than 200 N (Table 3). mFR did not differ by CNTF genotype for either force level.

The CNTF G/A and G/G group difference persisted with adjustments for multiple comparisons using false discovery rate control when considering all force levels, levels < 200 N, and levels >= 200 N. P values for false discovery rate control are given in the parentheses in Table 3. For all force levels, the first number represents an adjustment for 9 tests from our initial analyses which only included all force levels. The second p level is for all 21 tests considered for Table 3. For < 200 N and >= 200 N, only an adjustment for 21 tests is given, as this represented a post hoc analysis. Several interactions for all force levels tests lost their significant difference or showed a variable significant difference depending on the number of comparison tests.

**Figure 7:**
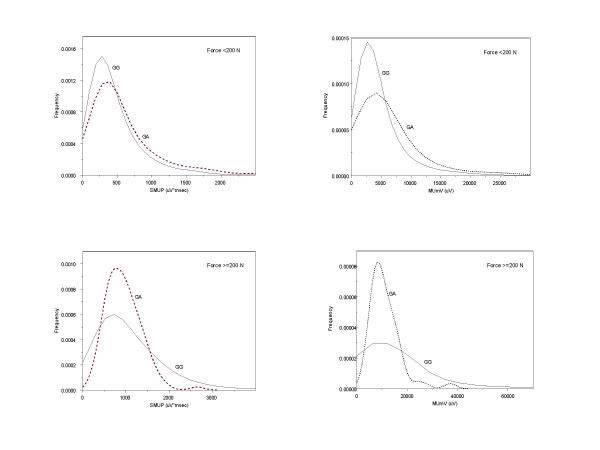
**Density distribution of motor unit size for CNTF GG and GA genotypes for force levels less than and greater than 200 N for SMUP and MUmV. **The graphs show the frequency distribution of motor units based on either SMUP or MUmV levels for genotypes GG and GA. The graphs show the interaction between CNTF genotype and motor unit properties.

To better demonstrate the differences in motor unit sizes between G/A and G/G at forces less than and greater than 200 N, density distributions were plotted for SMUP and MUmV (Figure 7). The density distributions for G/A and G/G differed for SMUP (p = 0) and MUmV (p = 0) for units less than 200 N, with a clear difference apparent with G/A being more dispersed with its mode at a higher SMUP and MUmV than G/G. For forces greater than 200 N, G/G is more dispersed than G/A for both SMUP (p = 0) and MUmV (p = 0).

### SMUP area

The G/A genotype was associated with activation of larger SMUP area at lower force levels and much smaller SMUP at levels above 200 N with a slower increase in SMUP area with increasing force output than the G/G genotype (Figure 1). A significant interaction was found between *CNTF *genotype by force when adjusting for age and gender (Table 3).

### mFR

mFR data revealed a significant interaction between force and CNTF genotype. Individuals with the G/A genotype were slower to increase mFR with increasing force than those from G/G subjects (Figure 2). No statistical difference or interaction was found between *CNTF *genotype and force (Table 3).

### MUmV

For G/A subjects, the MUmV was larger at low force levels, and smaller at high force levels than for G/G subjects (Figure 3). MUmV revealed a significant interaction between CNTF genotype by force similar to what was observed for SMUP area (Table 3).

### MURI

MURI, an index of the number of active units, was smaller at lower force levels and larger at higher force levels in G/A subjects than G/G subjects (Figure 4). A significant CNTF by force interaction was found (Table 3).

### SMUP area per unit force

The motor unit size (SMUP area) per unit force in G/A subjects declines with increasing force levels as would be expected with the progressive recruitment of larger motor units that are capable of generating greater force levels overall, as well as per MU fiber (Figure 5). In contrast, G/G subjects show a different pattern with little difference in motor unit size (SMUP area) per unit force at low and high force levels. SMUP area per unit force differed by force and CNTF genotype with a significant interaction (Table 3).

### MURI per unit force

At low force levels G/A subjects appear to use fewer motor units per unit force than G/G subjects (Figure 6). This is consistent with their use of larger units per unit force (Figure 1). At higher force levels, G/G and G/A subjects show the use of similar numbers of motor units per unit force. A significant interaction was found between CNTF genotype and force (Table 3).

## Discussion

Based on this preliminary study, CNTF genotype appears to influence the characteristics of the motor units of the vastus medialis active during submaximal knee extensor muscle contractions. G/A and G/G subjects appear to have different compositions of motor units that are reflected in the different sizes and firing patterns of motor units active during different levels of force generation. The strategy of motor unit activation appears to use Henneman's size principle with motor units becoming active based on their size. We speculate that G/A subjects show greater motor unit efficiency with increasing levels of force output than G/G individuals. G/A individuals appear to require less motor unit input per relative force level at higher force generation suggesting the utilization of smaller, potentially less fatigable units, which may be considered to be more efficient. At low force levels with the use of smaller, likely less fatigable motor units, G/A use fewer but larger units than G/G. Together the observations at low and high force levels suggest greater relative force generation per motor unit in participants of G/A genotype, which may lead to some loss of finer motor control in a muscle where the finest motor control may not be of prime importance. The force levels examined in this study are comparable to those used during daily activities and the differences in motor unit organization may have an impact on these activities.

In our previous report regarding CNTF and muscle strength, Roth et al [4] had found that G/A individuals were significantly stronger during isokinetic concentric testing at 3.14 rads/sec, but not at 0.52 rads/sec. In the current study, subjects were found not to differ in strength during isometric strength testing (i.e. 0 rads/sec). Roth et al [4] noted that the difference between 3.14 rads/sec and 0.52/sec suggested that the difference between G/G and G/A subjects may reflect differences in muscular power generation. The differences were not explained by differences in muscle mass, so Roth et al [4] suggested that the expressed null CNTF protein competed with the active CNTF protein for the CNTF receptor within muscle. How this would lead to increased strength was not clear. Muscular power is dependent on maximizing the use of strength and speed of movement, which is dependent on both the capability of muscle to generate force, and on the nervous system to maximize the coordination between force and speed. The observations in this report, while not directly addressing the power issue, suggest that neural organization in the peripheral motor system shows differences between G/A and G/G individuals that could result in differential maximal power generation.

CNTF is a gp130 cytokine member of the IL-6 superfamily that is present in both muscle and nerve. It appears to exert anabolic and catabolic effects that are important for muscle adaptation responses to injury [21]. In addition, the application of CNTF to female rats increases levator ani muscle volume and the number of muscle fibers, while not affecting muscle fiber size in younger rats [22]. CNTF administration in older rats resulted in a 17% increase in muscle fiber area [7]. CNTF also has known trophic, survival and regenerative effects on motor units [23], protects nerve conduction in diabetic rats [24], and is involved with motor neuronal sprouting [25]. In nerves, CNTF is predominantly localized to the Schwann cells of larger myelinated fibers [26,27], and enhances myelin formation [28]. These observations suggest that neural and myotrophic roles could explain the differences between G/A and G/G genotypes we observed in motor unit physiology. The differences between G/A and G/G subjects noted by Roth et al [4], and here, which could reflect differences in muscle power generation, may have potential importance in understanding the development of sarcopenia and frailty. Muscle power is related to functional disability in the elderly[29], and has been found to be related to longevity independent of isometric muscle strength[30].

The observations in this study are based on the motor unit properties as assessed using spiked triggered averaging and the decomposition program developed by Stashuk [13]. The methodology is based on approaches used to estimate the number of motor units in a muscle. Such methods give reasonable and reliable estimates of the number of motor units in a muscle [11]. We modified one of these approaches to examine the motor units during fixed levels of force generation during knee extension. The major advantage of the approach is the ability to study large numbers of subjects and to study them over time. In previous reports [14,15], we have noted limitations of the method. The main limitation stems from sampling bias. At any force level, the decomposition program is more likely to see larger than smaller active motor units. At low force levels this does not appear to be a problem. At higher levels, 30% and 50%, of MVC the program does not identify most small units (see Figure 1). However, the units being observed appear to account for most of the force generation, and are strongly related to the SEMG [19]. This issue should equally affect both G/G and G/A genotypes. However, the G/G subjects had smaller units at lower force levels, which if not observed would increase the regression estimate at higher force levels. The G/G subjects, in general had larger units at the higher force levels than did the G/A (Figure 1b). This can be seen by noting that in the 200 N to 450 N range, the 95th percentile for the G/A group was equivalent to the 81^st ^percentile for G/G group, while the G/A group actually had the smallest observed units in this force range. In this higher force range, the G/G group had both the smallest and largest recorded units. Any bias in unit selection does not appear to impact on G/G or G/A subjects in a way that would markedly alter the observations.

While CNTF genotype appears related to the pattern of motor unit activation in the vastus medialis, only one allele was studied, the A ("null") allele. Many genes are involved in the development, maintenance, and activation of the neural and muscular tissues that make up the motor unit. The observations in this study represent only a single component of this overall organization. A more thorough understanding of the process will require examining the interaction of a number of genes that are involved in motor unit activation.

## Conclusion

In summary, in this preliminary investigation, the presence of an inactive null allele in the CNTF gene resulted in a different pattern of motor unit activation during force generation between 10% and 50% of MVC compared to the most common CNTF genotype, force levels at which activities of daily living are performed. Subjects with the G/A genotype activated fewer but larger units at low force levels, and a greater number of relatively smaller units at higher force levels than G/G subjects. Whether the observation results in functional performance differences could not be determined from this study, however, could have important clinical implications and warrant further study at force levels used during daily activities.

## Methods

### Subjects

Two groups of subjects were used for the present analysis: 1) 36 women and men (aged 30 to 94 years) from the Baltimore Longitudinal Study of Aging [31], and 2) a cohort of 33 young and older men and women (23–73 years) who were evaluated prior to an exercise intervention study [32]. All subjects were healthy. Informed consent was obtained prior to each visit. Both studies were approved by the Johns Hopkins University Bayview Institutional Review Board.

### Strength

Maximal isometric force of the knee extensors was measured while participants were securely seated on the Kin-Com 125E isokinetic dynamometer (Chattecx, Chattanooga, TN) using methodology described previously [14,32]. Maximal voluntary isometric force in Newtons (N) was measured at a knee angle of 2.09 rad. Three maximal isometric contractions were performed with a ten second rest period between efforts. The average of the two best trials was used to represent MVC.

### Motor unit measurements

The methods for obtaining the motor unit measurements in the vastus medialis have been described in detail by Conwit et al. [14]. Subjects were tested while on the Kin-Com device as described above. The active surface detection electrode was placed over the motor point of the vastus medialis. To maximize the rise time of motor unit action potentials generated during low-level contractions, the concentric-needle electrode was inserted into the muscle body near the active surface electrode. Subjects then extended their knee against resistance with enough isometric force to achieve a specified percentage of their MVC. Visual and verbal cues were provided to the participant to help them achieve and maintain that level of force for 20–30 seconds. Simultaneously detected intramuscular and surface EMG signals were acquired using bandpass filtering from 10 Hz to 10 kHz and 5 Hz to 1000 Hz and sampling rates of 25 kHz and 2.5 kHz, respectively. The intramuscular EMG signal was decomposed using algorithms developed by Stashuk [13] to obtain accurate estimates of mean MU firing rates, and were combined with the surface EMG signal to provide estimates of surface-detected motor unit action potentials (SMUPs) using spike-triggered-averaging. After movement and readjustment of the position of the intramuscular electrode in order to minimize MU potential rise time, subjects were asked to generate similar force level contractions until SMUPs from 15–20 motor units were sampled at each force level studied. Previous reports using this method have shown that analysis of 15–20 motor units results in a coefficient of variation of approximately 10% [14]. Motor unit measurements were obtained during contraction levels of 10, 20, 30, and 50% of MVC for the Baltimore Longitudinal Study of Aging cohort, while measures were available only for 10 and 30% MVC for the exercise intervention cohort.

### Genotype

Genomic DNA was extracted from whole blood samples and CNTF genotype was determined as described previously [4]. Subjects were categorized as exhibiting the G/G, G/A, though no A/A homozygotes were observed in the present study.

### Data analysis

Mixed effects models were used to examine CNTF genotype differences in the relationship between motor unit variables with force generation while controlling for age, and gender. Mixed effects models are flexible models that can be used to deal with multiple data collected from groups or individuals as seen with longitudinal and repeated measures [33]. In the current study, each subject was tested at multiple force levels with approximately 15 motor units collected at each force level. The force levels were at 10, 20, 30 and 50% of MVC. Each subject may differ to some degree from the group as a whole, and this is dealt with by introducing random effects which were included for the intercept (i.e. how much the intercept from a subject's individual regression line deviated from the overall intercept), and for the percent of MVC) to allow for differences in how an individual responded to increasing force generation. All models had the following form

Y_ij _= (β0+b_j_0)+ β1*CNTF_j_+(β2+ b_j_2)*percent_j_+ β3*force_j_+ β4*CNTF_j _*force_j_+ β5*age_j_+ β6*gender_j_+ e_ij_

with Y_ij _being the motor unit property for the ith motor unit and jth subject and b_j_0 and b_j_2 being the random effect for subject j and percent j. All dependent variables were examined for deviation from normality. Terms that included SMUP were markedly skewed, and were log transformed for the analysis. We assumed that SMUP variance would increase with increasing force, as larger units are sequentially activated with increasing variance, and modeled variance as a power function in relationship to the percent of effort [33]. This assumption was found not to influence the reported findings.

Significant interactions were observed between CNTF and force, which suggested that at both higher and lower forces levels motor unit properties differed by genotype, with G/A using larger units at lower force levels. To directly test this hypothesis, the mixed effects model was applied only to data with force levels less than or greater/equal to 200 N. This force level was approximately where the curves for GA and GG intersected. In addition, density plots were graphed for SMUP and MUmV for GA and GG for force levels less than and greater/equal to 200 N. Differences between the GA and GG densities were tested using the Kolmogorov-Smirnov goodness-of-fit test.

At the initial submission, Table 3 contained 9 specific statistical tests which raise issues regarding the extent of need for consideration of multiple comparisons to control for falsely rejecting the null hypothesis. A traditional approach would be to adjust the significance level using the Bonferonni adjustment by dividing the test p by the number of tests to control type 1 or familywise error. However, this is an extremely conservative approach that increases the risk of accepting a false null hypothesis (type 2 error). An alternative approach is to control the false discovery rate, i.e. the expected proportion of type I errors among all significant tests. Attempting to hold this constant is different from the familywise error rate, where the goal is to avoid any type 1 errors [34]. We have added the adjusted p values to the models using the entire dataset and 2 adjustments, first for the 9 tests performed for Table 3 in the initial submission (3 of which were excluded in subsequent revisions), and for the 21 tests when including tests for force levels below and above 200 N.

Analyses were completed in SPLUS 6.2 (In Sightful, Seattle, WA). Chi-square and t-tests were used to compare subject characteristics. Data are means ± SE. Statistical significance was accepted at P < 0.05. Graphs show scatter plots with loess nonparametric regression lines to show the nature of the relationships between the motor unit variables and force generation [20].

## List of abbreviations used

CNTF: ciliary neurotrophic factor

mFR: mean firing rate

MUmV: motor unit mean voltage

MURI: motor unit relative index

MVC: maximal voluntary muscle contraction

SEMG: surface electromyography

SMUP: surface motor unit potential

## Authors' contributions

RAC and EJM developed the motor unit protocol that was used in the study. They were responsible for the collection of the motor unit data that was used in the analysis. They were the principal writers of the manuscript, and EJM did the analyses. SL was involved with the planning of the analyses and in the thinking that resulted in Table 1. She contributed to the manuscript preparation. SR and RF did the DNA work and with BH were responsible for initiating our interest in the impact of CNTF on muscle and motor unit function. All three contributed their expertises to the preparation of the manuscript. DS developed the spiked trigger averaging methodology, worked with RAC and EJM in developing and implementing the motor unit protocol, and contributed to the manuscript preparation.

## Figures and Tables

**Table 1:** Explanation of the assessed motor unit parameters

Measure	Calculation	Conversion-Units	Assessment
*Individual Motor Unit Measures*			
SMUP area	Surface motor unit potential area	uV*msec	Motor unit size
mFR	Mean firing rate	/sec	Motor unit mean firing rate
*Estimates of motor unit relationship to muscle activation*			
MUmV	(SMUP area)*FR	0.001* uV	Motor unit mean contribution to contraction
			
MURI	SEMG/1000*MUmV		Relative index of number of active units in field of measurement during contraction
*Estimate of overall muscle characteristics per unit force*			
(SMUP area)/N	Surface motor unit potential area per Newton force generated	uV*msec/N	Mean motor unit size per Newton force generated during a contraction
mFR/N	Mean firing rate per Newton force	1/sec*N	Mean motor unit firing rate per Newton force generated during a contraction
MUmV/N	(SMUP area)*mFR/N	uV/N	Mean motor unit contribution per Newton force
MURI/N	MURI/N	1000/N	Relative index of number of active units per unit force

**Table 2:** Subject Characteristics

	G/A	G/G	P
Sample size	12	58	
Age	52.0 (19.3)	51.2 (19.8)	0.89
Percent Female	42	33	0.35
Height	173.1 (9.5)	171.1 (7.8)	0.48
Weight	78.7 (14.2)	76.7 (18.1)	0.68
Isometric MVC	522.5 (160.9)	542.2 (173.6)	0.71

**Table 3:** Significance levels for mixed effects models examining force to motor unit relationships

Dependent Variables	All Force Levels			
	CNTF	Percent+	Force+	CNTF*Force

SMUP area*	0.01 (0.02, 0.02)	0.00	0.00	0.02 (0.06, 0.04)
mFR	0.32 (0.32,0.36)	0.01	.00	0.13 (0.14, 0.15)
MUmV*	0.00 (0.01, 0.02)	0.02	0.00	0.00 (0.06, 0.03)
MURI*	0.01 (0.02, 0.02)	0.07	0.013	0.01 (0.06, 0.03)4
(SMUP area)/force*	0.01 (0.02, 0.02)	0.00	.00	0.03 (0.07, 0.03)
MURI/force*	0.0 (0.00, 0.00)	0.00	0.00	0.05 (0.07, 0.07)
	Force < 200 N			
SMUP area*	0.00 (0.02)	0.07	0.00	0.01 (0.03)
mFR	0.37 (0.39)	0.10	0.00	0.23 (0.26)
MUmV*	0.00 (0.01)	0.19	0.00	0.00 (0.02)
MURI*	0.00 (0.02)	0.69	0.65	0.01 (0.03)
(SMUP area)/force*	0.01 (0.02)	0.09	0.59	0.03 (0.05)
MURI/force*	0.01 (0.02)	0.01	0.00	0.01 (0.03)
	Force > -200 N			
SMUP area*	0.01 (0.02)	0.22	0.00	0.00 (0.00)
mFR	0.57 (0.57)	0.45	0.00	0.96 (0.96)
MUmV*	0.03 (0.04)	0.42	0.00	0.00 (0.00)
MURI*	0.28 (0.32)	0.12	0.00	0.12 (0.15)
(SMUP area)/force*	0.01 (0.02)	0.15	0.00	0.00 (0.00)
MURI/force*	0.19 (0.24)	0.09	0.00	0.08 (0.12)

Each model was adjusted for age and gender. Numbers within parentheses are p values adjusted for multiple comparisons based on 9 and 21 tests for all force levels, and 12 tests for force< 200 N and force >= 200 N using the false discovery rate adjustment. *Variable was studied with natural log transform. + Percent refers to the force generated as a percent of MVC during motor unit collection. Force refers to the force generated during data collection.
